# Medium‐chain Acyl‐COA dehydrogenase deficiency: Pathogenesis, diagnosis, and treatment

**DOI:** 10.1002/edm2.385

**Published:** 2022-10-27

**Authors:** Emily Mason, Charles C. T. Hindmarch, Kimberly J. Dunham‐Snary

**Affiliations:** ^1^ Department of Biomedical and Molecular Sciences Queen's University Kingston Ontario Canada; ^2^ Queen's CardioPulmonary Unit Queen's University Kingston Ontario Canada; ^3^ Department of Medicine Queen's University Kingston Ontario Canada

**Keywords:** dietary management, fatty acids, medium‐chain Acyl‐CoA dehydrogenase, metabolic myopathy, β‐Oxidation

## Abstract

**Introduction:**

Medium‐Chain Acyl‐CoA Dehydrogenase Deficiency (MCADD) is the most common inherited metabolic disorder of β‐oxidation. Patients with MCADD present with hypoketotic hypoglycemia, which may quickly progress to lethargy, coma, and death. Prognosis for MCADD patients is highly promising once a diagnosis has been established, though management strategies may vary depending on the severity of illness and the presence of comorbidities.

**Methods and Results:**

Given the rapid developments in the world of gene therapy and implementation of newborn screening for inherited metabolic disorders, the provision of concise and contemporary knowledge of MCADD is essential for clinicians to effectively manage patients. Thus, this review aims to consolidate current information for physicians on the pathogenesis, diagnostic tools, and treatment options for MCADD patients.

**Conclusion:**

MCADD is a commonly inherited metabolic disease with serious implications for health outcomes, particularly in children, that may be successfully managed with proper intervention.

## Graphical abstract

MCADD is a commonly inherited metabolic disease with serious implications for health outcomes, particularly in children, that may be successfully managed with proper intervention. In this review, we discuss the genetics, pathogenesis, clinical presentation, diagnosis, and treatments for Medium‐Chain Acyl‐CoA Dehydrogenase Deficiency (MCADD), consolidating contemporary knowledge on MCADD to provide clinicians with adequate background and information to better understand and manage the illness in their patients.

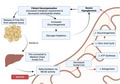

## 1 INTRODUCTION

β‐oxidation is the process of fatty acid metabolism, and in eukaryotes is performed in the mitochondria of cells. Medium‐Chain Acyl‐CoA Dehydrogenase (MCAD) is an enzyme key to the β‐oxidation of medium‐chain fatty acids (MCFAs).Medium‐Chain Acyl‐CoA Dehydrogenase Deficiency (MCADD) is the most common inherited metabolic disorder of β‐oxidation, in which patients have insufficient levels of functional MCAD.^1^ MCADD is typically diagnosed within the first two years of life and is characterized by hypoketotic hypoglycaemia and vomiting that may progress to seizures and coma in times of catabolic stress or prolonged fasting. MCADD is thought to be responsible for a small portion of sudden infant death syndrome (SIDS) cases worldwide, with an estimated prevalence of 1 in 50,000 births.^2^, ^3^

Mitochondria are key to the pathogenesis of MCADD. Mitochondria are membrane bound organelles found almost ubiquitously among eukaryotes. Mitochondria possess two lipid bi‐layer membranes (outer and inner)—this double membrane facilitates the performance of many key functions for survival of an organism, including ß‐oxidation and cellular respiration.^4^, ^5^

Several shuttle transport systems exist across the outer and inner membranes that are essential to the central functions of mitochondria. The glycerol 3‐phosphate (GP) shuttle connects mitochondrial and cytosolic processes, thereby playing a crucial role in cell bioenergetics.^6^ More significantly for the metabolism of fatty acids (FAs), the carnitine transport system is an important metabolic shuttle for the metabolism of long‐chain fatty acids (LCFAs). The carnitine transport system is crucial to β‐oxidation, allowing FAs to cross the inner mitochondrial membrane (IMM) and committing them to oxidation.^6^ A deficiency of MCAD may result in a build‐up of acylcarnitines in the urine and low serum carnitine concentrations, which may be observed in patients with biochemical testing.^7^ Importantly, FAs of carbon number up to C_8_ permeate the IMM passively, independent of the carnitine transport system.^8^ As such, function of β‐oxidative enzymes, including MCAD is dependent on functionality of this transport system for access to C_8_–C_12_ FA's.^9^

Mitochondrial β‐oxidation of saturated fatty acids is a central metabolic pathway for energy provision in an organism. Some tissues utilize the products of β‐oxidation for ketogenesis; the resultant ketone bodies are a crucial energy source in extrahepatic tissues, particularly under fasting conditions.^8^ The whole‐body response to fasting state, indicated by a low insulin: glucagon ratio, initiates lipolysis and ketogenesis.^10^ Simultaneously, glycogenolysis occurs for the rapid mobilization of glucose for use in extrahepatic tissue; the rationing of glucose through providing an alternate energy source in ketogenesis is crucial for the body to adequately respond to fasting conditions.^1^ Thus, β‐oxidation of FAs and its regulation are imperative for an organism's metabolic functioning, particularly in times of catabolic stress.

**FIGURE 1:**
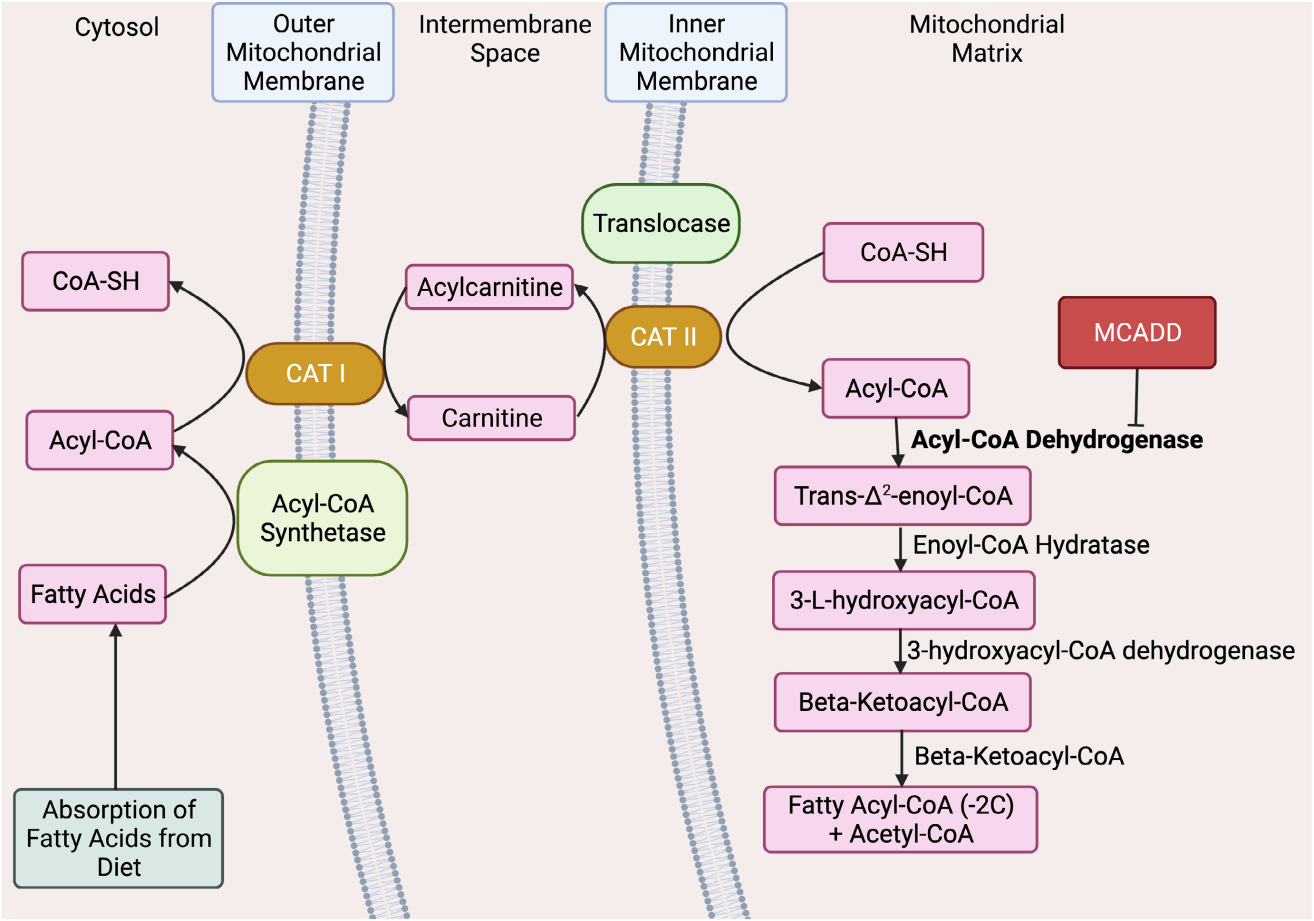
Beta‐Oxidation & its relation to MCADD. The metabolic pathway of MCFA oxidation is depicted including absorption, carnitine transport into the mitochondrial matrix, and ß‐oxidation. The normal activity of MCAD is bolded, and impact of MCAD deficiency indicated with a red box.

MCAD is an endogenous regulatory enzyme in FA β‐oxidation, specifically in the oxidation of MCFAs that arise from dietary triglycerides.^11^ MCAD catalyses the 2‐electron oxidation of fatty Acyl‐CoA thioesters to 2‐enoyl‐CoA, acting on MCFAs of carbon numbers C_6_ and C_12_ ^1^, ^8^; This is the first step of β‐oxidation. As an Acyl‐CoA dehydrogenase, MCAD is a key regulator of metabolic flux in β‐oxidation **(**Figure 1 **)**.

Individuals who survive initial presentation of MCADD access significantly more health resources in the first few years of life when compared with their healthy peers, and often manage life‐long challenges related to their condition.^12^ This review will therefore provide the reader with a comprehensive understanding of the clinical presentation, pathogenesis, and genetics of MCADD before discussing modern developments in the diagnosis and treatment that may provide improved patient outcomes.

## 2 CLINICAL PRESENTATION

**FIGURE 2:**
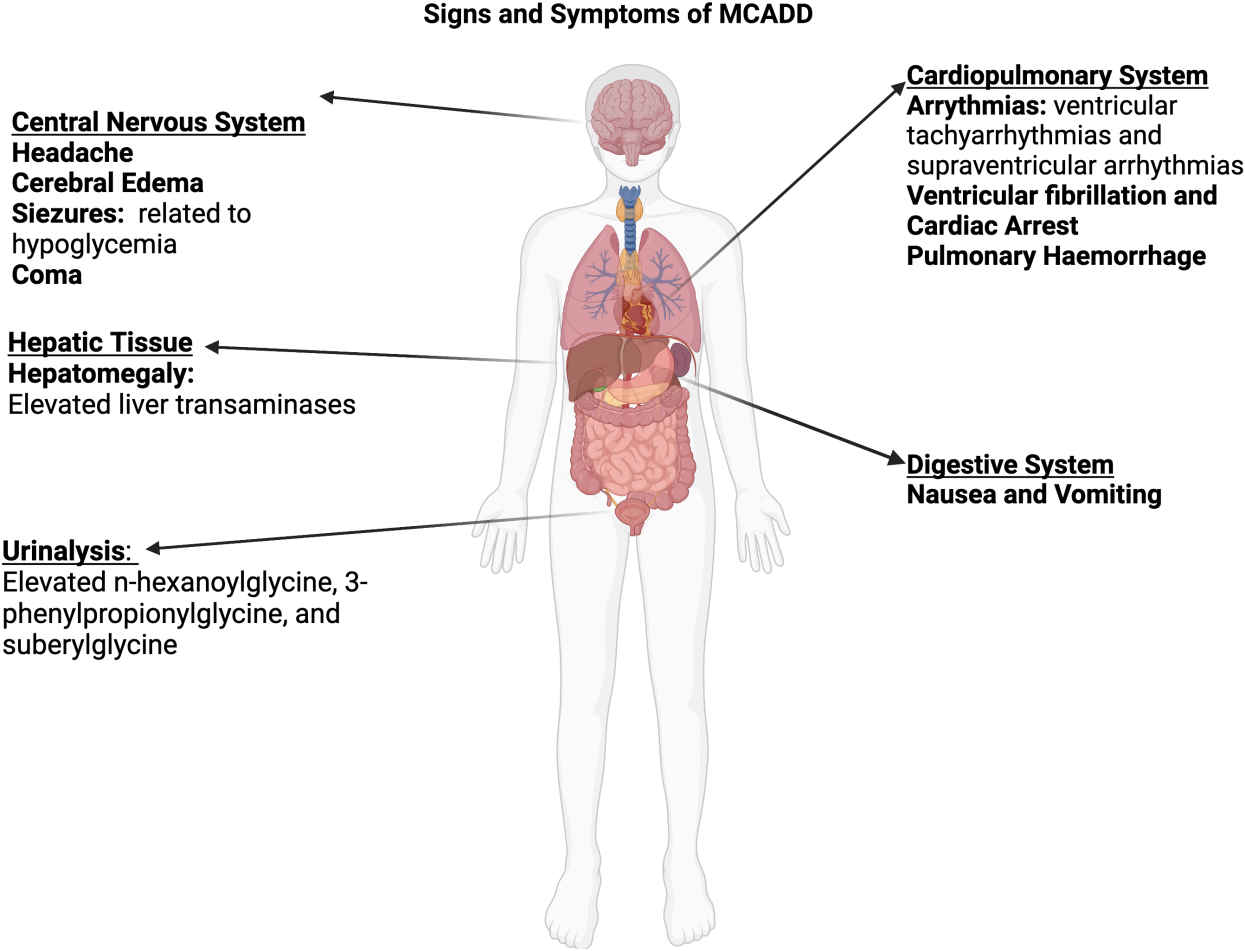
Signs and Symptoms of MCADD. The clinical presentation of MCAD deficiency is described along with the organ system most heavily impacted by each observed symptom.

Individuals with MCADD present as healthy at birth; first clinical presentation is typically within the first 24 months of life, though presentations up into adulthood have been reported. Presentation of symptoms typically coincides with incidence of prolonged fasting; in infancy, a reduction in overnight feedings can trigger onset of symptoms. Commonly, intercurrent or common infections will cause onset of symptoms in previously asymptomatic individuals.^13^ It is thought infection, particularly fever, increases metabolic requirements while simultaneously causing reduced appetite and calorie consumption, thus causing hypoglycaemia or other symptoms.^14^ A summary of documented symptoms is summarized in Figure 2.

Hypoglycaemic episodes are the characterizing feature of MCADD. Individuals may present with hypoglycaemia‐related seizures, which are indicative of severe metabolic stress, and may quickly progress to complete metabolic decompensation, vomiting, coma, and death.^14^ Hepatomegaly is a sign of severe, life‐threatening illness, and should therefore be considered an acute medical emergency.^15^ This is characterized by the presence of hypoketotic hypoglycaemia as discussed above and will likely be accompanied by lethargy. Initial laboratory results may also indicate increased anion gap, elevated liver transaminases and hyperammonaemia.^13^

Sudden death has historically been the first manifestation of MCADD in a portion of patients; up to 25% of MCADD‐affected individuals will die during their first clinical manifestation of the condition. MCADD is an established cause of sudden infant death syndrome; this is most common when infants display no defining features of MCADD or receive normal newborn screening results prior to the onset of illness.^2^, ^13^, ^16^, ^17^

Arrhythmias can be induced by the accumulation of medium‐chain acylcarnitines in MCADD, particularly in infancy. In neonatal cases, prolongation of the QT interval has also been reported.^18^, ^19^ Though relatively rare, ventricular tachyarrhythmias are the most commonly reported MCADD‐associated arrhythmia. Other reported arrhythmias include supraventricular arrhythmia and ultimately ventricular fibrillation resulting in cardiac arrest.^1^ Cardiac arrest is typically only observed after presentation with vomiting and headaches along with hyperammonaemia and hypoketotic hypoglycaemia. Isolated cases of pulmonary haemorrhage have also been observed.^20^ Lastly, it has been reported in individual cases that MCADD was discovered in infants following presentation with cardiomyopathy, though this has not been widely observed.^19^

## 3 PATHOGENESIS

**FIGURE 3:**
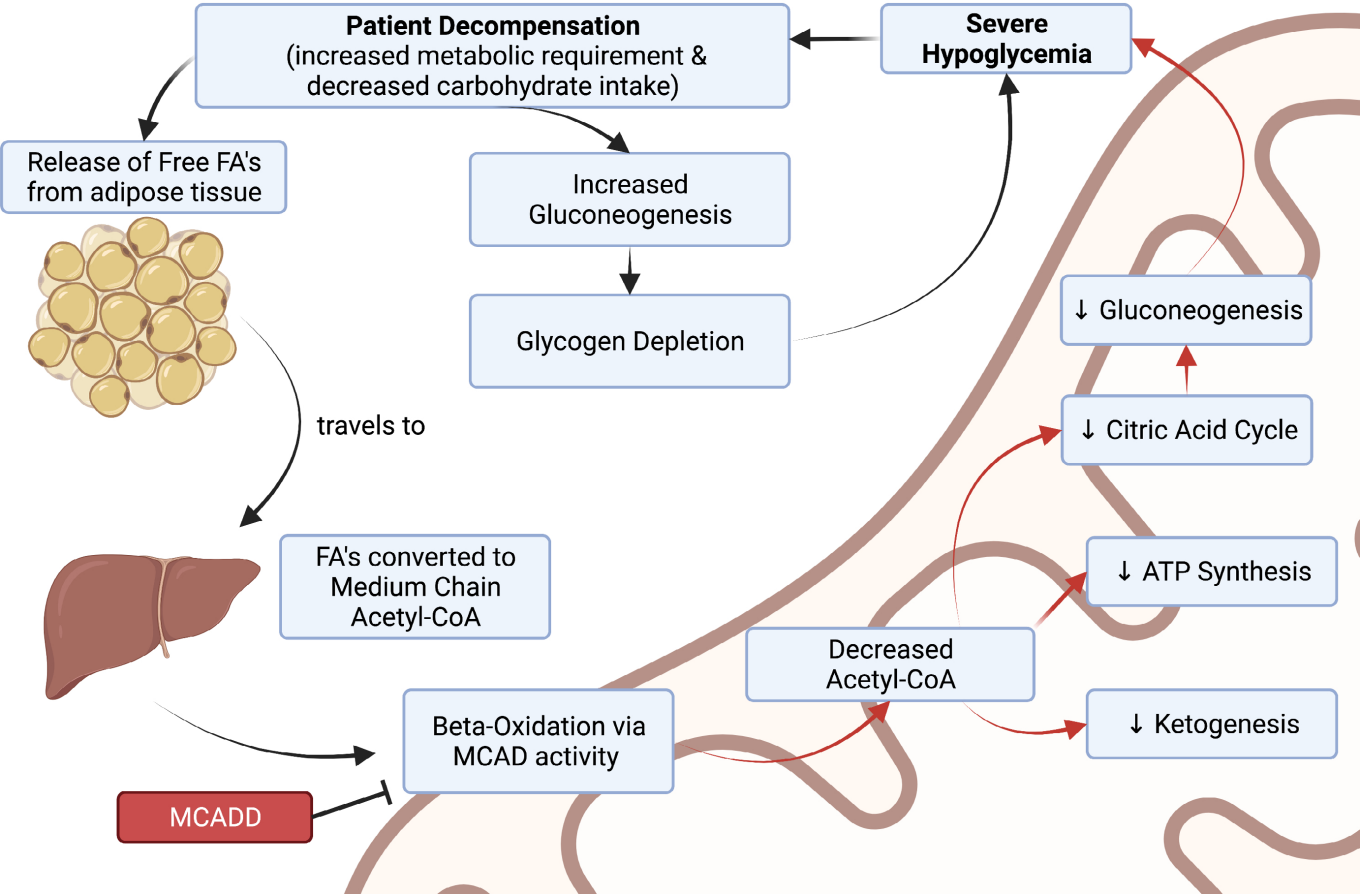
Mechanism of MCADD‐related patient decomposition. Physiological impact of MCADD on patient metabolism is indicated. Direct impacts of MCAD deficiency are indicated with red arrows.

Under normal conditions, MCADD patients compensate for a lack of energy production from β‐oxidation by expending glycogen stores elsewhere in the body. However, glycogen stores quickly become depleted in times of catabolic stress, specifically during illness and prolonged fasting.^13^ MCADD interferes with other significant metabolic pathways, preventing the production of ketone bodies and subsequent mobilization of glucose. Specifically, MCAD deficiency prevents the dehydrogenation step of β‐oxidation within the mitochondria resulting in decreased production of Acetyl‐CoA and a build‐up of acylcarnitines from the carnitine transport of MCFAs.^1^ Acetyl‐CoA is a requirement for ketogenesis to produce ketone bodies for energy usage in extrahepatic tissues.^4^ The reduction in energy sources exasperates the impact of reduced gluconeogenesis from a lack of carbohydrate intake; the body quickly progresses to severe hypoglycaemia, which in turn heightens patient decompensation.^13^ The depletion of glycogen, paired with an inability to utilize MCFAs to produce ketone bodies for energy, ultimately results in rapid progression to hypoglycaemia and metabolic decompensation **(**Figure 3 **)**.

In addition to the pathogenic consequences of Acetyl‐CoA deficiency from MCADD, secondary defects may contribute to MCADD‐associated symptoms. Evidence suggests that MCAD deficiency results in ineffective mitochondrial oxidative phosphorylation (OXPHOS); in vitro experimentation using MCAD‐deficient cell lines revealed impaired mitochondrial oxygen consumption and reduced levels of OXPHOS protein complexes.^21^ Decreased OXPHOS function is linked to reduced skeletal muscle function, such as muscle weakness that is often observed in MCADD affected individuals.^22^ It is theorized that the accumulation of MCFAs and their derivatives lead to dysfunction in neurological energy functions, resulting in the observed neurological complications such as encephalopathy.^23^

Altogether, the inability to metabolize MCFAs and subsequent metabolic imbalances in the form of Acetyl‐CoA deficiency and accumulation of MCFAs and acylcarnitines result in several pathogenic processes.

## 4 GENETICS

### 4.1 Genetic aetiology

MCADD is an autosomal recessive inherited metabolic disorder caused by mutations to the ACADM gene on chromosome 1p31, ultimately impacting the function of MCAD.^24^ Zhang et al first described the organization of the ACADM gene in 1992, demonstrating that the gene has 12 introns and spans 44 kb of DNA; the large size of the gene coupled with the number of introns results in a wide range of possible mis‐splicing events and other large mutations such as insertions or deletions.^25^ Fundamentally, it is understood that pathogenic mutations in the ACADM gene result in protein misfolding and subsequent loss of function.^26^

**FIGURE 4:**
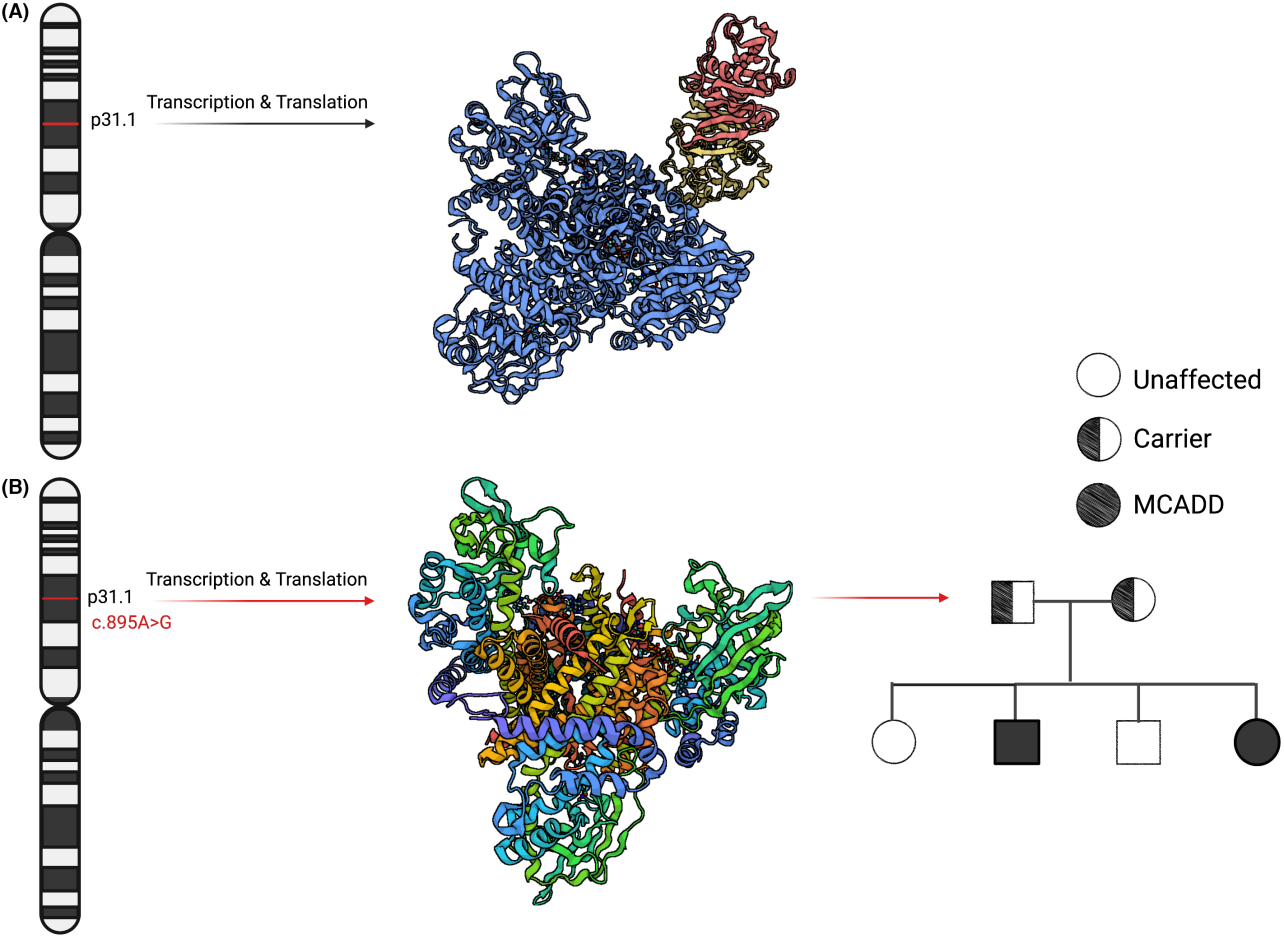
Genetic mutations of ACADM gene resulting in MCAD Protein Misfolding. (A) Normal protein structure of MCADD. The location of the ACADM gene is indicated on Chromosome 1 with a red line. The MCAD protein is depicted in blue, in complex with the electron transport chain (ETC). (B) Location, protein misfolding, and inheritance of K329E mutation. The structure of the MCAD protein harbouring the K329E mutation is depicted. The autosomal recessive inheritance pattern of the mutation is depicted in a pedigree chart on the right.

Approximately 60% of symptomatic homozygous patients present with a missense mutation in ACADM (c.985A > G) in which lysine is exchanged for glutamate at position 304, ultimately resulting in complete protein misfolding and loss of function (Figure 4 **).** ^27^ Most MCADD patients are either homozygous or heterozygous for c.985A > G; heterozygous patients may have a mild phenotype, indicating there may be other factors contributing to both severity of presentation and age of onset.^1^, ^28^ Individuals of Northern European descent have higher incidence, with an estimated prevalence of 80%, of the pathogenic c.985A > G mutation.^1^, ^29^ Tanaka et al. evaluated the mutations of 178 symptomatic MCADD patients in 1992, first reporting a 90% incidence of the K304E mutation and identifying three lesser prevalent mutations.^25^ Such mutations include the point mutation Tyr42His at an allelic frequency of 6%. Since the first reports of the genetic basis for MCADD, over 30 distinct mutations, the majority of which being missense mutations, have been identified in association with the pathology.^29^

### 4.2 Molecular genetic sequencing in diagnostics

Definitive diagnosis of MCADD requires sequencing of the ACADM gene for identification of mutations, as well as reduced MCAD activity in patient fibroblasts as determined through LC–MS/MS, typically ranging between 10% and 35% of regular enzyme activity.^30^, ^31^ Prenatal sequencing is possible in the case of maternal MCADD. Genetic testing may include a combination of gene‐targeted testing or comprehensive genomic testing; comprehensive genetic testing may reveal MCADD in patients who present at an older age without newborn screening (NBS) or for whom MCADD has not been considered as a diagnosis for symptoms.^1^

Gene‐targeted testing techniques like single‐gene testing or a multigene panel for ACADM is more likely in the case of a presumptive MCADD diagnosis following an abnormal elevation of acylcarnitines and other supportive biochemical results; single‐gene testing may also be used for patients who have heritage in an at‐risk population. Patients of Japanese descent should be screened for Japanese pathogenic variants, which account for 60% of alleles reported in the Japanese population: p.Thr150ArgfsTer4, p.Arg17His, p.Gly362Glu, and p.Arg53Cys.^32^ Similarly, patients of Northern European descent should undergo targeted analysis for European pathogenic variants: p.Lys329Glu and Tyr67His.Table 1 summarizes common genetic mutations in affected populations.^29^, ^32^, ^33^, ^34^, ^35^, ^36^, ^37^

**TABLE 1:** Common MCADD genetic mutations

Pathogenic variant nucleotide change	Type of mutation	Predicted protein change	High risk population	Disease presentation
c.985A > G	Missense	p.Lys329Glu	Northern European Descent	The most prevalent mutation causing MCADD, ~80% of patients with the c.895A > G are homozygous for the mutation, while 20% are heterozygous. Homozygous patients have highest levels of neonatal octanoylcartinine (C8).^11^
c.199 T > C	Missense	p.Tyr67His	Northern European Descent	The c.199 T > C mutation is less common, associated with less serious symptoms, and lower levels of plasma octoylcarnitines (C8). In some cases, this mutation is present without any sort of clinical presentation.^13^
c.449_452delCTGA	Deletion	p.Thr150ArgfsTer4	Japanese Descent	This mutation accounts for ~45% of cases in Japanese populations. Phenotypes range from asymptomatic to severe metabolic decompensation.^14^, ^15^
c.1085G > A	Missense	p.Gly362Glu	Japanese Descent	These four mutations account for ~60% of pathogenic variant alleles evaluated within the Japanese population.^16^, ^17^
c.50G > A	Missense	p.Arg17His	Japanese Descent	
c.157C > T	Missense	p.Arg53Cys	Japanese Descent	
c.843A > T	Missense	p.Arg281Ser	Japanese Descent	
c.727C > T	Missense	p.Arg243X	Chinese Descent	This mutation has only been reported among Chinese patients in mainland China, however no national‐scale epidemiology studies have been completed to elucidate the frequency of this variant in the wider Chinese population.^18^

### 4.3 Genetic counselling

Genetic counselling is available in some nations for relatives of affected individuals. Genetic sequencing is often recommended for first‐degree relatives upon the diagnosis of MCADD within a family with no prior known affected individuals, particularly given the autosomal recessive nature of disease inheritance.^1^, ^33^ These individuals may appear as asymptomatic, exhibiting no measurable differences in acylcarnitines, and as such require genetic sequencing for the exclusion of MCADD; nevertheless, testing should be completed on siblings of probands due to the largely unknown relationship between genotype and phenotype of MCADD, which may result in individuals remaining asymptomatic until a sudden onset later in life.^2^

Importantly, the link between genotype and phenotype is ambiguous at best; even well‐established pathogenic mutations like c.985 A > G display a wide range of symptoms and outcomes. Thus, it is supported that the disease is also dependent upon other inherited or environmental variables, though these factors remain to be established.^29^, ^38^

## 5 DIAGNOSIS

### 5.1 Newborn screening

NBS for MCADD was established in most ‘developed’ nations by the 1990's, although some jurisdictions, including the Canadian province of Ontario, did not introduce MCADD screening into regular NBS until as late as 2006.^39^, ^40^ The introduction of NBS has widened our understanding of both the epidemiology and genetic heterogeneity of the disease. Prior to NBS for MCADD, the incidence of the c.985A > G mutation was thought to be responsible for ~90% of cases worldwide, it is now understood to be the cause of only ~60% of cases as less common mutations have been identified.^29^ The number of children testing positive for known and novel MCADD‐associated mutations exceeded expected incidence, with many children being completely asymptomatic; this has allowed a more accurate evaluation of worldwide incidence rate, currently estimated between 1:18,000 and 1:20,000, higher in some European countries, including Denmark and England.^39^, ^40^, ^41^, ^42^, ^43^ Interestingly, presentation in infancy or diagnosis of MCADD from NBS has also allowed the identification of the disorder in mothers who had gone undiagnosed prior to presentation in their children.

That said, false‐positive results can arise from newborn screening testing. A 2016 study evaluating false positivity in a Canadian paediatric cohort found that patients who received a false‐positive test for MCADD had significantly more interactions with healthcare services in the first year of life compared with those who received true negative tests.^44^ This may indicate that the psychosocial impact of MCADD diagnoses may play a significant role in health behaviour and service utilization.

Medium chain length acylcarnitines, specifically octanoyl‐carnitine (C_8_) and decanoyl‐carnitine (C_10_) are measured on newborn screening blood spot cards using tandem mass spectrometry. C_8_ levels must be above a 0.40 μmol/L threshold to qualify for further testing and diagnosis, which is above the 99th percentile, while a triplicate of C_8_ levels of 0.50 μmol/L is often required for definitive diagnosis using NBS.^42^ Some evidence suggests that octanoylcarnitine levels may be impacted by a time lapse between time of sample collection and testing, so repetition of tests in the case of abnormal results is indicated and included in standard practice for most NBS protocols.^39^

Infants who display abnormally high levels of C_8_ or C_10_ should be referred to secondary treatment centers for definitive diagnosis or further genetic screening. Early diagnosis is preferable so as to establish long‐term management strategies and prevent symptom onset when feeding intervals lengthen, as well as reduce the likelihood of developmental delays and other complications associated with the disease.^45^

### 5.2 LC–MS/MS blood analysis of acylcarnitine

While NBS using dried blood samples has been successful in its identification of affected children, there is concern over its associated false positivity rate; LC–MS/MS has been suggested as an additional or alternative test to blood spot analysis to reduce the incidence of false‐positive tests.^46^

Liquid chromatography tandem mass spectrometry is the standard method for diagnosis of MCADD.^47^ Lymphocytes isolated from whole blood samples or newborn dried blood spots taken from the umbilical cord or heel stick are quantified via LC–MS/MS, in which C8:1‐CoA, C8:0‐CoA, and C8:OH‐CoA are isolated first with high pressure liquid chromatography and subsequently analysed for structure using tandem MS/MS.^48^ Patients with homozygous c.985A > G mutations consistently present with MCAD activity below 2.5%, while heterozygous or mild MCADD patients may range between 5.7 and 13.9% of normal activity, defined as 2.78 nmol/(min •mg protein).^45^ This method is the most applicable for widespread screening methods and has been implemented in newborn screening programs in developed nations.

### 5.3 Urinalysis

Asymptomatic individuals may show normal acylcarnitine levels using tandem mass spectrometry, and therefore require urine acylglycine assays to demonstrate elevated n‐hexanoylglycine (HG), 3‐phenylpropionylglycine, octanoylglycine (OG) and suberylglycine (SG).^1^ These assays are completed once again using LC–MS/MS, wherein HG has been identified as the most significant diagnostic marker for MCADD. Newer methods utilize ultra‐performance liquid chromatography tandem mass spectrometry (UPLC–MS/MS) to compare ratios of acylglycines; HG/acetylglycine (AG), OG/AG, and SG/OG have been demonstrated as excellent markers for MCAD deficiency.^30^, ^46^ Crucially, presence of ketones in preliminary urinalysis should not be taken as evidence against an MCADD diagnosis because acute metabolic decompensation may cause the presence of some ketones.^13^

### 5.4 Autopsy

Death in the first manifestation of MCADD for previously undiagnosed individuals has been reported in between 18 and 25% of cases.^49^ In these cases, results of urinalysis or NBS may not have been used to conclusively diagnose MCADD as an underlying cause of metabolic decompensation and as such autopsy may provide certainty to a cause of death. Findings at autopsy in the case of MCADD‐related sudden death commonly include cerebral oedema and fatty infiltration of the heart, liver, and kidneys.^49^, ^50^

### 5.5 Differential diagnoses

Reye‐like syndrome is characterized by acute metabolic encephalopathy caused by inborn errors of metabolism, MCADD among them. All causes of a Reye‐like syndrome should be considered in the differential diagnosis of MCADD. Reye's syndrome may be distinguished from MCADD by the age of onset; often, Reye's syndrome presents in older children with a history of aspirin ingestion.^9^ Other deficiencies in fatty acid β‐oxidation should be considered, specifically other disorders in the acyl‐dehydrogenase gene family like short‐chain‐specific Acyl‐CoA dehydrogenase, long‐chain‐specific Acyl‐CoA dehydrogenase, and very‐long‐chain‐specific Acyl‐CoA dehydrogenase. The presence of cardiomyopathy along with hypoketotic hypoglycaemia indicates long chain dehydrogenase deficiency.^51^ Deficiencies in any of these enzymes, or a combination of these enzymes as in Multiple Acyl‐CoA Dehydrogenase Deficiency (MADD), should be seriously evaluated before making a final diagnosis.^52^ Additionally, deficiencies in ketogenesis such as HMG‐CoA lyase deficiency may be considered.^53^ Ornithine transcarbamylase (OTC) deficiency, a rare X‐linked genetic disorder resulting in accumulation of ammonia, may result in similar clinical findings like lethargy and vomiting, though biochemical findings should display high concentrations of ammonia typical of urea cycle disorders.^54^

## 6 TREATMENT

### 6.1 Initial presentation

Management of MCADD varies depending on the severity and stage of the disease. In acute illness, patients are at high risk of experiencing metabolic crisis, which should be considered a medical emergency.^1^, ^55^ Reversal of catabolism and prevention of hypoglycaemia should be the immediate priority, and to this end simple carbohydrates may be given by mouth; provision of the substrates for glycolysis and subsequently the citric acid cycle, allows for sufficient energy production and symptom reversal. If the individual is unable to receive sufficient oral intake of glucose, IV administration of 2 ml/kg of 25% dextrose solution should be started immediately.^43^, ^56^ This should be followed by IV administration of 10% dextrose at 1.5 times the maintenance rate until a steady blood‐glucose reading of 5 mmol/L or higher is achieved; in the event that 25% dextrose solution is unavailable, administration of 10% dextrose as described may be sufficient.^55^, ^57^, ^58^

### 6.2 Long‐term management

The therapeutic goal of long‐term MCADD management is to prevent acute incidents of metabolic decompensation through avoidance of fasting. In times of normal ‘health’ and the absence of an intercurrent infection with fever or stressing conditions, fasting times should be kept to a minimum.^59^ Avoidance of fasting is of particular importance in times of illness when MCADD patients are at high risk for metabolic crisis. Patients who follow a strict feeding regimen have successfully prevented the onset of hypoketotic hypoglycaemia or other MCADD symptoms.^51^ The provision of a medical alert bracelet may speed intervention should an acute incident require emergency care.

### 6.3 L‐carnitine supplementation

Dietary supplementation of L‐carnitines is controversial.^60^ L‐carnitines have historically been prescribed as a supplement to MCADD patients as part of a long‐term management plan at a dosage of 100 mg/kg/day; some patients require a lower dose of 50 mg/kg/day due to the side effect of fish‐smelling body odour resulting from the drug.^61^, ^62^ It has been theorized that L‐carnitine supplementation may alleviate the secondary carnitine deficiency from acylcarnitine ester accumulation.^62^, ^63^ The benefits of such supplementation remain unclear and are largely unverified.^64^, ^65^, ^66^ One study evaluating the impact of L‐carnitine supplementation at 50 mg/kg/day on performance of moderate intensity exercise found that while the carnitine group had significantly higher plasma concentrations of octanoylcarnitine, suggesting that supplemented patients had increased clearance of accumulated acylcarnitines, both groups had elevated free carnitine levels in plasma and urine; thus, they found no significant biochemical advantage for carnitine supplementation in MCADD patients.^61^ Further, L‐carnitine supplements have been shown to have no preventative effect on the development of complications or comorbidities such as obesity.^64^

### 6.4 Development of comorbidities

MCADD‐affected individuals are at risk of developing related conditions; long‐term management should include regular screening for symptoms by a physician. Evidence suggests that individuals with MCADD and similar FA metabolic disorders are at higher risk of developing chronic renal disease as they age, namely fatty infiltration of the kidney and tubulointerstitial fibrosis.^59^

It is recommended that dietary management be supervised by a general physician or dietician. In addition to avoidance of fasting, dietary management should include avoidance of foods and infant formula that contain medium chain fatty acids. MCADD patients are at high risk of developing obesity as a side effect of increasing their number of meals, so ensuring proper balance of nutrition is important.^1^, ^59^ In addition to obesity, the development of diabetes mellitus II has been reported in a number of patients who were diagnosed in infancy and followed an MCADD‐specific diet; managing both diabetes mellitus II along with MCADD presents a unique challenge and should be managed under the supervision of an endocrinologist.^67^

Muscular concerns are prominent in individuals who have experienced uncontrolled metabolic decompensation with their MCADD.^68^ Chronic muscle weakness, fatigue, pain, and reduced exercise tolerance have been reported; interestingly, no abnormality in cardiac function is associated with these symptoms.^13^ Physical therapy in consultation with a physician may be indicated in patients who develop muscular concerns following an episode of MCADD symptoms.

Additionally, MCADD patients with first clinical presentation in infancy are at higher risk of developing neurologic conditions from uncontrolled metabolic decompensation. Loss of developmental milestones have been reported, as have been aphasia and attention deficit disorder (ADD).^69^ ADD is thought to be caused by brain injury during acute metabolic crisis, and is associated with disordered eating patterns and obesity.^13^ MCADD affected individuals have been reported to be at risk for eating disorders such as anorexia nervosa.^70^ In the case of neurological findings, support and treatment by mental health professionals is recommended.

### 6.5 Considerations for adult patients

Some patients may not present with a hypoglycaemic episode associated with MCADD well into adulthood. While adult presentations of MCADD are rare, many occur after recent ingestion of alcohol and subsequent vomiting; it is thought that alcohol ingestion was accompanied by prolonged fasting.^68^ Interestingly, cardiac symptoms are reported more frequently in adult patients than their younger counterparts. This may be due to prolonged uptake of free MCFA's into cardiomyocytes through childhood and adolescence, ultimately resulting in cardiovascular damage or arrhythmias.^68^, ^71^

## 7 TREATMENTS IN DEVELOPMENT

Currently, there are no cures or enzyme‐replacement treatments available for MCADD. This is an area of research of particular interest considering the high incidence and long‐term burden for both the affected individual and the healthcare sector. Development of several experimental treatments have been initiated in the last number of years including pharmacological intervention and gene therapy, though comparisons of intervention efficacy across different trials is significantly hindered by different groups utilizing different outcome measures. Moving forward, the inclusion of core outcome measures in MCADD therapeutic clinical trials would be of great value, particularly given the challenges of gathering large cohorts for rare diseases such as MCADD.^72^, ^73^

Currently, the drug glycerol phenylbutyrate, called Ravicti® (Clinical Trial Identifier: NCT02246218), has been evaluated as an experimental treatment for MCADD patients to improve MCAD stability and reduce metabolite build‐up. Ravicti® has previously been FDA approved for use in urea cycle disorders and has recently finished Phase I clinical trials at the University of Pittsburgh for the treatment of MCADD and related urea cycle disorders.^74^ As a nitrogen scavenger, it is thought that Ravicti® may help address the secondary hyperammonaemia that occurs in some severe MCADD cases, often in the presence of hepatomegaly and supraventricular tachycardia.^1^, ^74^, ^75^ The clinical study was severely limited by sample size, restricting their ability to perform many analyses evaluating the efficacy of the drug. Primary results were promising in reducing hyperammonaemia in children aged less than 2 months, though use of glycerol phenylbutyrate is not a widely used intervention for MCADD affected children as of the time of this publication.^76^

As with many inborn errors of metabolism, gene therapy provides an exciting avenue for treatment development, particularly given evidence that even moderate increase in enzyme function can improve patient outcomes. In situ gene therapies have been explored for MCADD, focused on skeletal muscle tissue as the primary location of β‐oxidation.^77^ While there are a variety of pathogenic mutation types that result in MCADD ranging from single‐nucleotide polymorphisms (SNPs) to large deletions, the majority of symptomatic individuals harbour single point mutations. Thus, a precise gene editing technique allowing for base‐to‐base conversion, for example the use of a prime editor for targeted mutagenesis without double stranded DNA breaks, may represent an exciting avenue of therapeutic development for clinically relevant mutations like c.985A > G.^78^

In vitro and in vivo animal studies have been performed using recombinant adenovirus (rAD) vectors as a potential for clinical use, but no human clinical trials have been published using this vector for gene therapy.^79^ Recent work evaluating the role of secondary defects in oxidative phosphorylation on MCADD used the lentiviral‐based Crisper/Cas9 system to create an MCAD knockout cell line; while helpful in elucidating mechanisms of MCADD pathogenesis, no published studies have used this system in a therapeutic capacity.^21^ These cell models would provide an invaluable resource for future preclinical studies. This is a rapidly developing field and is of particular interest for new MCADD treatments.

## 8 CONCLUSION

MCADD is the most common inherited disease of fatty acid oxidation, impacting patients worldwide, and is sometimes lethal despite the introduction of widespread newborn screening.^29^ MCADD is most commonly characterized by hypoketotic hypoglycaemia in the context of prolonged fasting or common illness.^15^ It may be initially treated with glucose supplementation orally or via IV. The gold standard for diagnosis is LC–MS/MS as part of NBS, and genetic testing is sometimes recommended to identify the specific mutation causing the condition.^30^ Long‐term management of MCADD by dietary management is largely successful, though physician monitoring for development of related conditions should be continued in times of relative health.^1^ Future study should include the development of alternative therapeutic interventions to address complications managing dietary intake; gene therapy using adenovirus vectors provides a unique therapeutic avenue for comprehensive treatment and should therefore be a focus of future study.^79^

## AUTHOR CONTRIBUTIONS

**Emily Mason:** Conceptualization (lead); writing – original draft (lead); writing – review and editing (supporting). **Charles C. T. Hindmarch:** Writing – review and editing (equal). **Kimberly J. Dunham‐Snary:** Conceptualization (equal); funding acquisition (lead); supervision (lead); writing – review and editing (lead).

## CONFLICT OF INTEREST

Authors have no competing interests to declare.

## ETHICS APPROVAL & PATIENT CONSENT

N/A.

## Data Availability

No datasets were generated or analysed during the current study.

## References

[1] Merritt JL 2nd , Chang IJ . Medium‐chain Acyl‐coenzyme a dehydrogenase deficiency. In: Adam MP , Ardinger HH , Pagon RA , et al., eds. GeneReviews([R]). Mayo Clinic; 1993.

[2] McConkie‐Rosell A , Iafolla AK . Medium‐chain acyl CoA dehydrogenase deficiency: its relationship to SIDS and the impact on genetic counseling. J Genet Couns. 1993;2:17‐27. 24242229 10.1007/BF00962557

[3] Rocha H , Castineiras D , Delgado C , et al. Birth prevalence of fatty acid beta‐oxidation disorders in Iberia. JIMD Rep. 2014;16:89‐94. 25012579 10.1007/8904_2014_324 PMC4221301

[4] Friedman JR , Nunnari J . Mitochondrial form and function. Nature. 2014;505:335‐343. 24429632 10.1038/nature12985 PMC4075653

[5] Wang C , Youle R . Cell biology: form follows function for mitochondria. Nature. 2016;530:288‐289. 26887490 10.1038/530288a

[6] Martinez J , Marmisolle I , Tarallo D , Quijano C . Mitochondrial bioenergetics and dynamics in secretion processes. Front Endocrinol (Lausanne). 2020;11:319. 32528413 10.3389/fendo.2020.00319 PMC7256191

[7] Couce ML , Sanchez‐Pintos P , Diogo L , et al. Newborn screening for medium‐chain acyl‐CoA dehydrogenase deficiency: regional experience and high incidence of carnitine deficiency. Orphanet J Rare Dis. 2013;8:102. 23842438 10.1186/1750-1172-8-102 PMC3718718

[8] Papamandjaris AA , MacDougall DE , Jones PJ . Medium chain fatty acid metabolism and energy expenditure: obesity treatment implications. Life Sci. 1998;62:1203‐1215. 9570335 10.1016/s0024-3205(97)01143-0

[9] Ibrahim S , Temtem T . Medium‐Chain Acyl‐COA Dehydrogenase Deficiency. StatPearls; 2021. 32809672

[10] Vakifahmetoglu‐Norberg H , Ouchida AT , Norberg E . The role of mitochondria in metabolism and cell death. Biochem Biophys Res Commun. 2017;482:426‐431. 28212726 10.1016/j.bbrc.2016.11.088

[11] Houten SM , Violante S , Ventura FV , Wanders RJ . The biochemistry and physiology of mitochondrial fatty acid beta‐oxidation and its genetic disorders. Annu Rev Physiol. 2016;78:23‐44. 26474213 10.1146/annurev-physiol-021115-105045

[12] Karaceper MD , Khangura SD , Wilson K , et al. Health services use among children diagnosed with medium‐chain acyl‐CoA dehydrogenase deficiency through newborn screening: a cohort study in Ontario, Canada. Orphanet J Rare Dis. 2019;14:70. 30902101 10.1186/s13023-019-1001-0 PMC6431026

[13] Gartner V , McGuire PJ , Lee PR . Child neurology: medium‐chain acyl‐coenzyme a dehydrogenase deficiency. Neurology. 2015;85:e37‐e40. 26215884 10.1212/WNL.0000000000001786 PMC4520810

[14] Baruteau J , Levade T , Redonnet‐Vernhet I , Mesli S , Bloom MC , Broue P . Hypoketotic hypoglycemia with myolysis and hypoparathyroidism: an unusual association in medium chain acyl‐CoA desydrogenase deficiency (MCADD). J Pediatr Endocrinol Metab. 2009;22:1175‐1177. 20333879 10.1515/jpem.2009.22.12.1175

[15] Baruteau J , Sachs P , Broue P , et al. Clinical and biological features at diagnosis in mitochondrial fatty acid beta‐oxidation defects: a French pediatric study of 187 patients. J Inherit Metab Dis. 2013;36:795‐803. 23053472 10.1007/s10545-012-9542-6

[16] Yusupov R , Finegold DN , Naylor EW , Sahai I , Waisbren S , Levy HL . Sudden death in medium chain acyl‐coenzyme a dehydrogenase deficiency (MCADD) despite newborn screening. Mol Genet Metab. 2010;101:33‐39. 20580581 10.1016/j.ymgme.2010.05.007

[17] Gong Z , Liang L , Qiu W , et al. Clinical, biochemical, and molecular analyses of medium‐chain Acyl‐CoA dehydrogenase deficiency in Chinese patients. Front Genet. 2021;12:577046. 33841490 10.3389/fgene.2021.577046 PMC8025081

[18] Janeiro P , Jotta R , Ramos R , et al. Follow‐up of fatty acid beta‐oxidation disorders in expanded newborn screening era. Eur J Pediatr. 2019;178:387‐394. 30617651 10.1007/s00431-018-03315-2

[19] Marci M , Ajovalasit P . Medium‐chain Acyl‐CoA dehydrogenase deficiency in an infant with dilated cardiomyopathy. Cardiol Res Pract. 2009;2009:281389. 20049317 10.4061/2009/281389 PMC2796442

[20] Staels W , D'Haese J , Sercu E , De Meirleir L , Colpaert J , Cornette L . Medium‐chain Acyl‐CoA dehydrogenase deficiency presenting with neonatal pulmonary haemorrhage. Matern Health Neonatol Perinatol. 2015;1:8. 27057325 10.1186/s40748-015-0010-9 PMC4823675

[21] Lim SC , Tajika M , Shimura M , et al. Loss of the mitochondrial fatty acid beta‐oxidation protein medium‐chain Acyl‐coenzyme a dehydrogenase disrupts oxidative phosphorylation protein complex stability and function. Sci Rep. 2018;8:153. 29317722 10.1038/s41598-017-18530-4 PMC5760697

[22] Korzeniewski B . Effects of OXPHOS complex deficiencies and ESA dysfunction in working intact skeletal muscle: implications for mitochondrial myopathies. Biochim Biophys Acta. 2015;1847:1310‐1319. 26188374 10.1016/j.bbabio.2015.07.007

[23] Bennett MJ . Pathophysiology of fatty acid oxidation disorders. J Inherit Metab Dis. 2010;33:533‐537. 20824345 10.1007/s10545-010-9170-y

[24] Matsubara Y , Narisawa K , Tada K . Medium‐chain acyl‐CoA dehydrogenase deficiency: molecular aspects. Eur J Pediatr. 1992;151:154‐159. 1601002 10.1007/BF01954373

[25] Tanaka K , Yokota I , Coates PM , et al. Mutations in the medium chain acyl‐CoA dehydrogenase (MCAD) gene. Hum Mutat. 1992;1:271‐279. 1363805 10.1002/humu.1380010402

[26] Maier EM , Gersting SW , Kemter KF , et al. Protein misfolding is the molecular mechanism underlying MCADD identified in newborn screening. Hum Mol Genet. 2009;18:1612‐1623. 19224950 10.1093/hmg/ddp079 PMC2667288

[27] Yokota I , Indo Y , Coates PM , Tanaka K . Molecular basis of medium chain acyl‐coenzyme a dehydrogenase deficiency. An a to G transition at position 985 that causes a lysine‐304 to glutamate substitution in the mature protein is the single prevalent mutation. J Clin Invest. 1990;86:1000‐1003. 2394825 10.1172/JCI114761 PMC296821

[28] Curtis D , Blakemore AI , Engel PC , et al. Heterogeneity for mutations in medium chain acyl‐CoA dehydrogenase deficiency in the UK population. Clin Genet. 1991;40:283‐286. 1756601 10.1111/j.1399-0004.1991.tb03097.x

[29] Grosse SD , Khoury MJ , Greene CL , Crider KS , Pollitt RJ . The epidemiology of medium chain acyl‐CoA dehydrogenase deficiency: an update. Genet Med. 2006;8:205‐212. 16617240 10.1097/01.gim.0000204472.25153.8d

[30] Fisher L , Davies C , Al‐Dirbashi OY , Ten Brink HJ , Chakraborty P , Lepage N . A novel method for quantitation of acylglycines in human dried blood spots by UPLC‐tandem mass spectrometry. Clin Biochem. 2018;54:131‐138. 29402417 10.1016/j.clinbiochem.2018.01.020

[31] Bouvier D , Vianey‐Saban C , Ruet S , Acquaviva C . Development of a tandem mass spectrometry method for rapid measurement of medium‐ and very‐long‐chain Acyl‐CoA dehydrogenase activity in fibroblasts. JIMD Rep. 2017;35:71‐78. 27943070 10.1007/8904_2016_22 PMC5585096

[32] Tajima G , Hara K , Tsumura M , et al. Screening of MCAD deficiency in Japan: 16years' experience of enzymatic and genetic evaluation. Mol Genet Metab. 2016;119:322‐328. 27856190 10.1016/j.ymgme.2016.10.007

[33] Smith EH , Thomas C , McHugh D , et al. Allelic diversity in MCAD deficiency: the biochemical classification of 54 variants identified during 5 years of ACADM sequencing. Mol Genet Metab. 2010;100:241‐250. 20434380 10.1016/j.ymgme.2010.04.001

[34] Ehrnhoefer DE . MCAD mutations identified in newborn screening cause different levels of enzymatic dysfunction. Clin Genet. 2009;76:146‐148. 19673949 10.1111/j.1399-0004.2009.01247_1.x

[35] Purevsuren J , Kobayashi H , Hasegawa Y , et al. A novel molecular aspect of Japanese patients with medium‐chain acyl‐CoA dehydrogenase deficiency (MCADD): c.449‐452delCTGA is a common mutation in Japanese patients with MCADD. Mol Genet Metab. 2009;96:77‐79. 19064330 10.1016/j.ymgme.2008.10.012

[36] Hara K , Tajima G , Okada S , et al. Significance of ACADM mutations identified through newborn screening of MCAD deficiency in Japan. Mol Genet Metab. 2016;118:9‐14. 26947917 10.1016/j.ymgme.2015.12.011

[37] Li Y , Zhu R , Liu Y , Song J , Xu J , Yang Y . Medium‐chain acyl‐coenzyme a dehydrogenase deficiency: six cases in the Chinese population. Pediatr Int. 2019;61:551‐557. 31033143 10.1111/ped.13872

[38] Gregersen N , Andresen BS , Corydon MJ , et al. Mutation analysis in mitochondrial fatty acid oxidation defects: exemplified by acyl‐CoA dehydrogenase deficiencies, with special focus on genotype‐phenotype relationship. Hum Mutat. 2001;18:169‐189. 11524729 10.1002/humu.1174

[39] Rhead WJ . Newborn screening for medium‐chain acyl‐CoA dehydrogenase deficiency: a global perspective. J Inherit Metab Dis. 2006;29:370‐377. 16763904 10.1007/s10545-006-0292-1

[40] Kennedy S , Potter BK , Wilson K , et al. The first three years of screening for medium chain acyl‐CoA dehydrogenase deficiency (MCADD) by newborn screening Ontario. BMC Pediatr. 2010;10:82. 21083904 10.1186/1471-2431-10-82 PMC2996355

[41] Andresen BS , Lund AM , Hougaard DM , et al. MCAD deficiency in Denmark. Mol Genet Metab. 2012;106:175‐188. 22542437 10.1016/j.ymgme.2012.03.018

[42] Oerton J , Khalid JM , Besley G , et al. Newborn screening for medium chain acyl‐CoA dehydrogenase deficiency in England: prevalence, predictive value and test validity based on 1.5 million screened babies. J Med Screen. 2011;18:173‐181. 22166308 10.1258/jms.2011.011086 PMC3243649

[43] Maguolo A , Rodella G , Dianin A , et al. Diagnosis, genetic characterization and clinical follow up of mitochondrial fatty acid oxidation disorders in the new era of expanded newborn screening: a single Centre experience. Mol Genet Metab Rep. 2020;24:100632. 32793418 10.1016/j.ymgmr.2020.100632 PMC7414009

[44] Karaceper MD , Chakraborty P , Coyle D , et al. The health system impact of false positive newborn screening results for medium‐chain acyl‐CoA dehydrogenase deficiency: a cohort study. Orphanet J Rare Dis. 2016;11:12. 26841949 10.1186/s13023-016-0391-5 PMC4741015

[45] Vargas CR , Ribas GS , da Silva JM , et al. Selective screening of fatty acids oxidation defects and organic acidemias by liquid chromatography/tandem mass spectrometry Acylcarnitine analysis in Brazilian patients. Arch Med Res. 2018;49:205‐212. 30119976 10.1016/j.arcmed.2018.08.004

[46] Minkler PE , Stoll MSK , Ingalls ST , Hoppel CL . Correcting false positive medium‐chain acyl‐CoA dehydrogenase deficiency results from newborn screening; synthesis, purification, and standardization of branched‐chain C8 acylcarnitines for use in their selective and accurate absolute quantitation by UHPLC‐MS/MS. Mol Genet Metab. 2017;120:363‐369. 28190699 10.1016/j.ymgme.2017.02.006

[47] Nada MA , Chace DH , Sprecher H , Roe CR . Investigation of beta‐oxidation intermediates in normal and MCAD‐deficient human fibroblasts using tandem mass spectrometry. Biochem Mol Med. 1995;54:59‐66. 7551818 10.1006/bmme.1995.1009

[48] Tan J , Chen D , Huang J , Chang R , Yan T , Cai R . Tandem mass spectrometry analysis and genetic diagnosis of neonates with fatty acid oxidation disorders in central and northern regions of Guangxi. Zhonghua Yi Xue Yi Chuan Xue Za Zhi. 2019;36:1067‐1072. 31703127 10.3760/cma.j.issn.1003-9406.2019.11.003

[49] Yang Z , Lantz PE , Ibdah JA . Post‐mortem analysis for two prevalent beta‐oxidation mutations in sudden infant death. Pediatr Int. 2007;49:883‐887. 18045290 10.1111/j.1442-200X.2007.02478.x

[50] Bennett MJ , Rinaldo P , Millington DS , Tanaka K , Yokota I , Coates PM . Medium‐chain acyl‐CoA dehydrogenase deficiency: postmortem diagnosis in a case of sudden infant death and neonatal diagnosis of an affected sibling. Pediatr Pathol. 1991;11:889‐895. 1775402 10.3109/15513819109065485

[51] Yamada K , Taketani T . Management and diagnosis of mitochondrial fatty acid oxidation disorders: focus on very‐long‐chain acyl‐CoA dehydrogenase deficiency. J Hum Genet. 2019;64:73‐85. 30401918 10.1038/s10038-018-0527-7

[52] Prasun P . Multiple Acyl‐CoA dehydrogenase deficiency. In: Adam MP , Ardinger HH , Pagon RA , et al., eds. GeneReviews([R]). Mayo Clinic; 1993.

[53] Maduemem KE . Medium‐chain acyl‐coenzyme a dehydrogenase deficiency (MCADD): a cause of severe hypoglycaemia in an apparently well child. BMJ Case Rep. 2016;2016:bcr2016217538. 10.1136/bcr-2016-217538 PMC5174908 27903579

[54] Lichter‐Konecki U , Caldovic L , Morizono H , Simpson K , Ah Mew N , MacLeod E . Ornithine Transcarbamylase deficiency. In: Adam MP , Mirzaa GM , Pagon RA , et al., eds. GeneReviews([R]). Mayo Clinic; 1993.

[55] McGregor TL , Berry SA , Dipple KM , Hamid R , Council On G . Management principles for acute illness in patients with medium‐chain Acyl‐coenzyme a dehydrogenase deficiency. Pediatrics. 2021;147:e2020040303. 33372121 10.1542/peds.2020-040303

[56] Piercy H , Machaczek K , Ali P , Yap S . Parental experiences of raising a child with medium chain Acyl‐CoA dehydrogenase deficiency. Glob Qual Nurs Res. 2017;4:2333393617707080. 28516128 10.1177/2333393617707080 PMC5419063

[57] Ventura FV , Leandro P , Luz A , et al. Retrospective study of the medium‐chain acyl‐CoA dehydrogenase deficiency in Portugal. Clin Genet. 2014;85:555‐561. 23829193 10.1111/cge.12227

[58] Tong F , Jiang PP , Yang RL , et al. Medium‐chain acyl‐CoA dehydrogenase deficiency: neonatal screening and follow‐uP. Zhongguo Dang Dai Er Ke Za Zhi. 2019;21:52‐57. 30675864 10.7499/j.issn.1008-8830.2019.01.010 PMC7390178

[59] Schatz UA , Ensenauer R . The clinical manifestation of MCAD deficiency: challenges towards adulthood in the screened population. J Inherit Metab Dis. 2010;33:513‐520. 20532824 10.1007/s10545-010-9115-5

[60] Spiekerkoetter U , Bastin J , Gillingham M , Morris A , Wijburg F , Wilcken B . Current issues regarding treatment of mitochondrial fatty acid oxidation disorders. J Inherit Metab Dis. 2010;33:555‐561. 20830526 10.1007/s10545-010-9188-1

[61] Huidekoper HH , Schneider J , Westphal T , Vaz FM , Duran M , Wijburg FA . Prolonged moderate‐intensity exercise without and with L‐carnitine supplementation in patients with MCAD deficiency. J Inherit Metab Dis. 2006;29:631‐636. 16972171 10.1007/s10545-006-0355-3

[62] Madsen KL , Preisler N , Orngreen MC , et al. Patients with medium‐chain acyl‐coenzyme a dehydrogenase deficiency have impaired oxidation of fat during exercise but no effect of L‐carnitine supplementation. J Clin Endocrinol Metab. 2013;98:1667‐1675. 23426616 10.1210/jc.2012-3791

[63] Stanley CA . Carnitine deficiency disorders in children. Ann N Y Acad Sci. 2004;1033:42‐51. 15591002 10.1196/annals.1320.004

[64] Rinaldo P , Schmidt‐Sommerfeld E , Posca AP , Heales SJ , Woolf DA , Leonard JV . Effect of treatment with glycine and L‐carnitine in medium‐chain acyl‐coenzyme a dehydrogenase deficiency. J Pediatr. 1993;122:580‐584. 8463904 10.1016/s0022-3476(05)83539-5

[65] Jager EA , Schaafsma M , van der Klauw MM , Heiner‐Fokkema MR , Derks TGJ . Plasma carnitine concentrations in medium‐chain acyl‐CoA dehydrogenase deficiency: lessons from an observational cohort study. J Inherit Metab Dis. 2022. doi:10.1002/jimd.12537. Online ahead of print. PMC9796739 35778950

[66] Yokoi K , Ito T , Maeda Y , et al. Acylcarnitine profiles during carnitine loading and fasting tests in a Japanese patient with medium‐chain acyl‐CoA dehydrogenase deficiency. Tohoku J Exp Med. 2007;213:351‐359. 18075239 10.1620/tjem.213.351

[67] Afreh‐Mensah D , Agwu JC . Coexistence of medium chain acyl‐CoA dehydrogenase deficiency (MCADD) and type 1 diabetes (T1D): a management challenge. BMJ Case Rep. 2021;14:e239325. 10.1136/bcr-2020-239325 PMC7993300 33762273

[68] Lang TF . Adult presentations of medium‐chain acyl‐CoA dehydrogenase deficiency (MCADD). J Inherit Metab Dis. 2009;32:675‐683. 19821147 10.1007/s10545-009-1202-0

[69] Landau Z , Pinhas‐Hamiel O . Attention deficit/hyperactivity, the metabolic syndrome, and type 2 diabetes. Curr Diab Rep. 2019;19:46. 31250219 10.1007/s11892-019-1174-x

[70] Rohr F . Nutrition management of fatty acid oxidation disorders. In: Bernstein L , Rohr F , Helm J , eds. Nutrition Management of Inherited Metabolic Diseases. Springer; 2015:271‐282.

[71] Derks TG , Reijngoud DJ , Waterham HR , et al. The natural history of medium‐chain acyl CoA dehydrogenase deficiency in The Netherlands: clinical presentation and outcome. J Pediatr. 2006;148:665‐670. 16737882 10.1016/j.jpeds.2005.12.028

[72] Pugliese M , Tingley K , Chow A , et al. Outcomes in pediatric studies of medium‐chain acyl‐coA dehydrogenase (MCAD) deficiency and phenylketonuria (PKU): a review. Orphanet J Rare Dis. 2020;15:12. 31937333 10.1186/s13023-019-1276-1 PMC6961328

[73] Potter BK , Hutton B , Clifford TJ , et al. Establishing core outcome sets for phenylketonuria (PKU) and medium‐chain Acyl‐CoA dehydrogenase (MCAD) deficiency in children: study protocol for systematic reviews and Delphi surveys. Trials. 2017;18:603. 29258568 10.1186/s13063-017-2327-3 PMC5735866

[74] Kormanik K , Kang H , Cuebas D , Vockley J , Mohsen AW . Evidence for involvement of medium chain acyl‐CoA dehydrogenase in the metabolism of phenylbutyrate. Mol Genet Metab. 2012;107:684‐689. 23141465 10.1016/j.ymgme.2012.10.009 PMC3504130

[75] Palir N , Ruiter JPN , Wanders RJA , Houtkooper RH . Identification of enzymes involved in oxidation of phenylbutyrate. J Lipid Res. 2017;58:955‐961. 28283530 10.1194/jlr.M075317 PMC5408614

[76] Longo N , Diaz GA , Lichter‐Konecki U , et al. Glycerol phenylbutyrate efficacy and safety from an open label study in pediatric patients under 2 months of age with urea cycle disorders. Mol Genet Metab. 2021;132:19‐26. 33388234 10.1016/j.ymgme.2020.12.002 PMC8655853

[77] Schowalter DB , Matern D , Vockley J . In vitro correction of medium chain acyl CoA dehydrogenase deficiency with a recombinant adenoviral vector. Mol Genet Metab. 2005;85:88‐95. 15896652 10.1016/j.ymgme.2005.02.006

[78] Park SJ , Jeong TY , Shin SK , et al. Targeted mutagenesis in mouse cells and embryos using an enhanced prime editor. Genome Biol. 2021;22:170. 34082781 10.1186/s13059-021-02389-w PMC8173820

[79] Keeler AM , Flotte TR . Cell and gene therapy for genetic diseases: inherited disorders affecting the lung and those mimicking sudden infant death syndrome. Hum Gene Ther. 2012;23:548‐556. 22642257 10.1089/hum.2012.087 PMC3392613

